# Secure Transmission in mmWave Wiretap Channels: On Sector Guard Zone and Blockages

**DOI:** 10.3390/e21040427

**Published:** 2019-04-22

**Authors:** Yi Song, Weiwei Yang, Zhongwu Xiang, Yiliang Liu, Yueming Cai

**Affiliations:** 1 College of Communications Engineering, Army Engineering University of PLA, No. 88 Houbiaoying, Qinhuai District, Nanjing 210007, China; 2 School of Physics and Electronic Electrical Engineering, Huaiyin Normal University, Huai’an 223300, China; 3 Communications Research Center, Harbin Institute of Technology, Harbin 150001, China

**Keywords:** physical layer security, millimeter wave, sector secrecy guard zone, artificial noise

## Abstract

Millimeter-wave (mmWave) communication is one of the key enabling technologies for fifth generation (5G) mobile networks. In this paper, we study the problem of secure communication in a mmWave wiretap network, where directional beamforming and link blockages are taken into account. For the secure transmission in the presence of spatially random eavesdroppers, an adaptive transmission scheme is adopted, for which sector secrecy guard zone and artificial noise (AN) are employed to enhance secrecy performance. When there exists no eavesdroppers within the sector secrecy guard zone, the transmitter only transmits information-bearing signal, and, conversely, AN along with information-bearing signal are transmitted. The closed-form expressions for secrecy outage probability (SOP), connection outage probability (COP) and secrecy throughput are derived under stochastic geometry. Then, we evaluate the effect of the sector secrecy guard zone and AN on the secrecy performance. Our results reveal that the application of the sector secrecy guard zone and AN can significantly improve the security of the system, and blockages also can be utilized to improve secrecy performance. An easy choice of transmit power and power allocation factor is provided for achieving higher secrecy throughput. Furthermore, increasing the density of eavesdroppers not always deteriorates the secrecy performance due to the use of the sector secrecy guard zone and AN.

## 1. Introduction

In recent years, data traffic increases significantly with the rapid popularization of various mobile intelligent devices and the growth of wireless data, and millimeter wave (mmWave) communication is an especially promising approach to meet the data traffic demand in the 5G and beyond wireless communication system because of the abundant available bandwidth of mmWave frequency [1,2]. There have been plenty of works presented in terms of achievable rate and coverage for mmWave communication system [3,4,5]. However, due to the wireless characteristic of electromagnetic wave and the openness of wireless channel, security remains a challenge to the design of mmWave systems. In this vein, there has been a heightened interest for safeguarding complex wireless networks by physical layer security (PLS).

The main idea of PLS is to make use of the normal randomness of wireless communication channel to guarantee that the confidential information is transferred to the legitimate receiver and that the confidential information will not be decoded by illegal users [6,7,8,9,10]. Reference [11] provided a detailed, transparent and accurate information on the latest developments in the use of collaborative techniques to improve PLS. In addition, different cooperation technologies were classified, and their merits and demerits were discussed. It showed that the design of PLS schemes was still an important research field in 5G networks security. Recently, various technologies, like multiple-antenna, wiretap coding and signal processing technologies [12,13,14], especially guard zone and artificial noise (AN) [15,16], have become effective methods to enhance PLS. Using AN to enhance the reliability of legitimate links and interfere with eavesdropping links, so as to enlarge the gap between legitimate links and eavesdropping links to improve security. For different network applications, References [17,18] proposed the secrecy enhancement by using secrecy protected zone and AN, and discussed the relationship among protected zone, the transmission power and AN. In [19], the secrecy guard zone protocol was studied for achieving the secure transmission in an underlay cognitive radio network. However, all the aforementioned works on secrecy guard zone are only considered in the conventional microwave networks; they can not be directly applied and need to be re-evaluated in an mmWave system because of the unique characteristics of the mmWave communication system.

PLS in mmWave systems has attracted interest with enthusiasm [20,21,22,23]. The characteristics of mmWave communication system, such as larger bandwidth, large antenna arrays, directionality and short range transmission, could provide stronger PLS for mmWave system. Using analog beamforming in the mmWave base station, the secrecy throughput was analyzed from the perspectives of delay-tolerant and delay-limited transmissions in [24]. Considering the characteristics of mmWave cellular networks, Referecne [25] studied the secrecy performance of the noise-limited and the AN-assisted mmWave networks under the stochastic geometry framework. Referecne [26] examined the impact of AN on the secrecy rate; it was shown that power allocation between the information signal and AN need to be carefully determined for secrecy performance enhancement. A discrete angular domain channel model considering spatial discernibility path was proposed in [27], and three secure transmission schemes were investigated by depending on whether there was a common path between the destination and the eavesdropper. Although many insightful conclusions have been drawn in [25,27,28], the effects of blockages and the information leakage problem of the side lobe are not considered, but they are assumed to be ignored. In fact, blockages have different effects on communications in different environments, and side lobe may also lead to information leakage. On the other hand, directional beamforming is an important technique for mmWave systems because it provides array gains which overcome the huge path loss and acquire adequate link margins [4]. For mathematical tractability [29], when the simple maximum signal-to-noise ratio (SNR) beam steering is assumed, it is meaningful to approximate the actual array pattern with the sector pattern, where the directional gains of the main lobe and the side lobe are constant. The approximation of the sector mode makes it possible to describe the complex beamforming mode. Nevertheless, the locations of eavesdroppers in mmwave wiretap channels are randomly distributed, thus they may be located in signal beams and then could intercept confidential information. However, for a mmWave wiretap network, comprehensive secrecy performance analysis has not been provided under a sector secrecy guard zone, which motivates our work.

In this paper, we investigated secrecy performance under the Nakagami fading channel in a mmWave wiretap network. In order to improve the secrecy performance of mmWave wiretap network, a secrecy guard zone is introduced around the transmitter, in which eavesdroppers are not allowed to roam. It is assumed that the eavesdroppers can be detected, provided that they enter secrecy guard zone. Considering a more practical mmWave communication scenario, the effects of blockage are taken into account such that links are either line-of-sight (LOS) or non-line-of-sight (NLOS). In our prior conference paper [30], we discussed how to use sector guard zone in mmWave networks. Based on our previous work, assuming the transmitter is capable of detecting the existence of eavesdroppers in the finite guard zone, an adaptive transmission scheme is adopted for secrecy transmission. Our diversified contributions and insights are listed as follows:

- According to the characteristics of mmWave beam pattern, both the main lobe and side lobe are taken into consideration. Specifically, a sector secrecy guard zone model is considered to achieve theoretical design and analysis. Depending on the locations of eavesdroppers detected by a transmitter, an adaptive transmission scheme is proposed which chooses two types of transmission strategies adaptively. The fist-type is direct transmission when there exists no eavesdroppers in the sector secrecy guard zone and the second-type is the AN assisted transmission when one or more eavesdroppers in the sector secrecy guard zone.
- Stochastic geometry is adopted in proposed mmWave wiretap network to characterize the random spatial locations of eavesdroppers. The closed-form expressions of secrecy outage probability (SOP),connection outage probability (COP) and secrecy throughput are derived in the proposed scheme. In addition, we provide a further insight of the system parameters, i.e., transmit power, power allocation factor, secrecy guard zone radius and central angle, blockage density, antenna gain, and the intensity of the eavesdroppers into secrecy performance.
- The results show that enlarging the radius of sector secrecy guard zone improves secrecy performance. In addition, recruiting AN also enhances secrecy performance especially when the density of eavesdroppers is dense. In addition, blockage plays an important role in the transmission of mmWave, which can be utilized to improve secrecy performance. Furthermore, in our adaptive transmission scheme, increasing the density of eavesdroppers not always deteriorates the secrecy performance. Ultimately, simulations provide an easy choice of transmit power and power allocation factor for achieving higher secrecy throughput.

The remainder of this paper is organized as follows. Section 2 presents the system model and the performance metric. Section 3 introduces the secure transmission strategies. Section 4 derives the expressions of secrecy performance for adaptive transmission scheme. Numerical and simulation results verified our theoretical analysis are presented in Section 5. Finally, we conclude this paper in Section 6.

## 2. System Description and Performance Metrics

### 2.1. System Description

**Figure 1:**
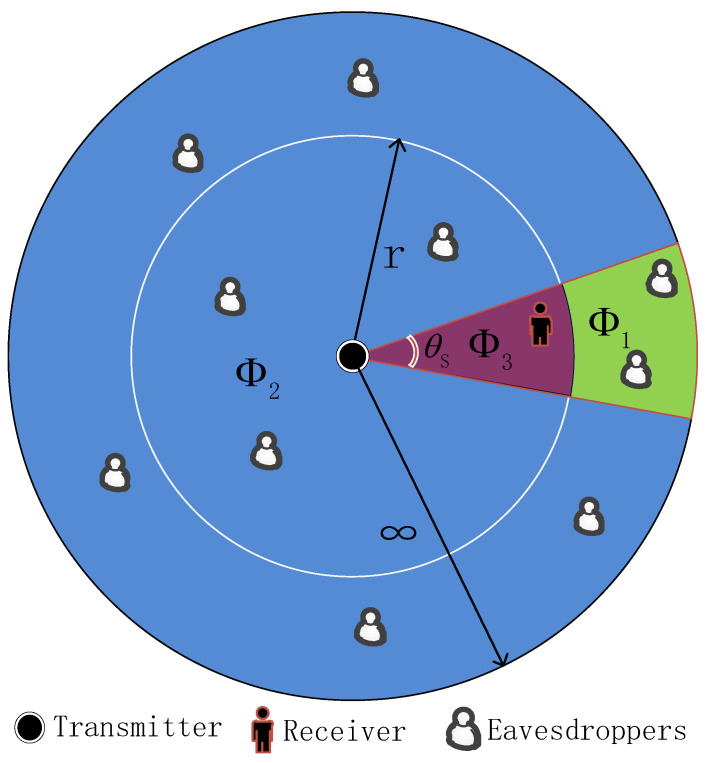
Network topology for the considered mmWave wiretap network. A sector secrecy guard zone is employed to approximate the beamforming pattern. $\Phi_{1}$, $\Phi_{2}$, $\Phi_{3}$ indicate the different areas where the eavesdroppers are located.

Let’s consider a mmWave wiretap network, which consists of a transmitter, a legitimate receiver and multiple random distributed eavesdroppers, as shown in Figure 1. The transmitter equipped with *M* multiple antennas uses directional beamforming for transmitting the confidential information. Both the legitimate receiver and the eavesdroppers equip a single antenna [31]. Furthermore, the locations of eavesdroppers, denoted by $\Phi_{E}$, are modeled as an independent homogeneous Poisson point process (HPPP) with density $\lambda_{E}$. Without loss of generality, similar to [32], a sector model is used to analyze the beam pattern in this paper, particularly

(1) $$G_{S}\left(\theta \right)=\left\{\begin{array}{cc} M_{S}, & if\left|\theta \right|\leq \theta_{S},\\ m_{S}, & otherwise, \end{array}\right.$$

where $M_{S}$ represents the main lobe gain with the beam width $\theta_{S}$, and $m_{S}$ represents the array gain of side lobe. We assume that the transmitter can get the perfect channel state information (CSI) of the legitimate receiver; then, they can trim their antenna steering orientation array to their legitimate receiver and maximize the directivity gains. In practical terms, estimating the CSI may be a nontrivial task, so our work actually provides an upper bound on achievable secrecy performance. In this model, the eavesdroppers have been in the attempt to intercept the confidential information of the system, the CSIs of eavesdroppers are assumed to be unknown at the transmitter. The nearest eavesdropper is not necessarily the most detrimental one, but the one possessing the best channel to the transmitter. In addition, we consider non-colluding eavesdroppers in this work.

The secrecy performance of the system is further improved by using the sector secrecy guard zone and AN. It is assumed that the eavesdroppers can be detected by scanning nearby eavesdropping devices before transmission [17,18], provided that they are close enough to the transmitter. Therefore, a sector secrecy guard zone having a radius of *r* is introduced, and the eavesdroppers may be in or out of the sector secrecy guard zone. Therefore, a sector secrecy guard zone having a radius of *r* is introduced, and the eavesdroppers may be in or out of the sector secrecy guard zone. Similar to the secrecy guard zone mechanism in Referecne [17], considering the characteristics of mmWave beam pattern, we model the finite range around the transmitter as a sector secrecy guard zone with radius *r* and central angle $\theta_{S}$. Considering the generalized fading environment, mmWave communication channel is modeled as a Nakagami fading model. It is different from [33], which studied the secrecy performance of random multiple-input multiple- output (MIMO) wireless networks based on homogeneous Poisson point process (HPPP) over the α-μ fading channel. It is worth pointing out that the estimation of mmWave channel is more consistent with the actual communication system, but it is beyond the scope of this paper.

According to the characteristics of mmWave in an outdoor scenario, the confidential information reaches to the legitimate receiver may be via LOS or NLOS [26]. According to 3GPP standards and the blockage model with random shape theory [34], the probability of a LOS with distance $r_{d}$ is represented by $P_{L}\left(r_{d}\right)$, while the NLOS probability is $P_{N}\left(r_{d}\right)$. The probability $P_{L}\left(r_{d}\right)$ and $P_{N}\left(r_{d}\right)$ are given as $P_{L}\left(r_{d}\right)=e^{-\beta r_{d}}$ or $P_{N}\left(r_{d}\right)=1-e^{-\beta r_{d}}$, which can be acquired from stochastic blockage models or field measurements, where β is the blockage density.

In light of the pass-loss model and small-scale fading presented in [35], the channel gain received by the legitimate receiver can be expressed as $M_{S}{\left|h_{D}\right|}^{2}L\left(r_{D}\right)$ and the eavesdroppers can be expressed as $M_{S}{\left|h_{E}\right|}^{2}L\left(r_{E}\right)$ or $m_{s}{\left|h_{E}\right|}^{2}L\left(r_{E}\right)$, where both ${\left|h_{D}\right|}^{2}$ and ${\left|h_{E}\right|}^{2}$ are normalized Gamma random variable with following ΓNL,11NLNL or ΓNN,11NNNN, $r_{D}$ and $r_{E}$ denote the distance from the transmitter to the legitimate receiver and the distance from the transmitter to the eavesdropper, $N_{L},N_{N}$ are the Nakagami fading parameter of LOS and NLOS, respectively. $L\left(r_{D}\right)$ and $L\left(r_{E}\right)$ denote the path loss function which are modeled as $L\left(r_{j}\right)=C_{L}r_{j}^{-\alpha_{L}}$ or $C_{N}{r_{j}}^{-\alpha_{N}}$, $j\in \left\{D,E\right\}$, $r_{j}$ is the distance in meters, $C_{L}$ and $\alpha_{L}$ are the constant and path loss exponent depending on the LOS, $C_{N}$ and $\alpha_{N}$ depend on the NLOS.

Based on the aforementioned adaptive transmission scheme, there are serious security threats when there exist one or more eavesdroppers in the sector secrecy guard zone. In order to enhance the secure transmission performance, using the superposition coding [18], the transmitter transmits information-bearing signals and sends AN, namely AN assisted transmission. If there exists no eavesdroppers in the sector secrecy guard zone, the transmitter still transmits useful information signals, namely direct transmission. As previously assumed, the locations of eavesdroppers are randomly distributed, the probability of no eavesdropper existed within the sector secrecy guard zone is given by $p_{e1}=\exp\left(-\theta_{S}\lambda_{E}r^{2}/2\right)$, and the probability of eavesdropper existed within the sector secrecy guard zone is given by $p_{e2}=1-\exp\left(-\theta_{S}\lambda_{E}r^{2}/2\right)$ [36].

### 2.2. Performance Metrics

In the following, we use the SOP, the COP and the secrecy throughput to measure secrecy performance [37].

#### 2.2.1. Secrecy Outage Probability and Connection Outage Probability

If the perfect security of confidential information can not be guaranteed, that is, a portion of the confidential information sent from the transmitter is decoded by at least one eavesdropper, the secrecy outage event takes place, the SOP is written as

(2) $$p_{so}=\mathrm{Pr}\left(\gamma_{E}>2^{R_{B}-R_{S}}-1\right),$$

where $\gamma_{E}$ denotes signal-to-interference-plus-noise received by the eavesdropper. Adopting Wyner code, $R_{S}$ and $R_{B}$ are the confidential information rate and codeword transmission rate, respectively, where $R_{B}\geq R_{S}$ [15,38].

If the confidential information cannot be decoded without error at the legitimate receiver, the connection outage event occurs, then the COP can be expressed as

(3) $$p_{co}=\mathrm{Pr}\left(\gamma_{D}<2^{R_{B}}-1\right),$$

where $\gamma_{D}$ denotes signal-to-interference-plus-noise received by the legitimate receiver.

#### 2.2.2. Secrecy Throughput

The secrecy throughput represents the average secrecy rate when information is both secure and reliably transmitted. When $p_{co}$ and $p_{so}$ are independent of each other, the secrecy throughput is given by [19]

(4) $$\eta =\left(1-p_{co}\right)\left(1-p_{so}\right)R_{S}.$$

## 3. Secure Transmission Strategies

In this section, we focus on the SNR received by the receiver and eavesdroppers in the mmWave wiretap network. We assume that the transmitter is able to detect the existence of eavesdroppers within the sector secrecy guard zone. We first analyze that the eavesdroppers are not in the sector secrecy guard zone, and then consider the eavesdroppers in the sector secrecy guard zone. It is worth mentioning that whether there are eavesdroppers in the sector secrecy guard zone or not, the transmitter transmits useful information signals. When the eavesdroppers are in the sector secrecy guard zone, we further exploit AN and produce positive effects through power adjustment control.

### 3.1. Eavesdroppers Are Detected Beyond the Sector Secrecy Guard Zone

If there do not exist eavesdroppers within the sector secrecy guard zone, the transmitter keeps sending the confidential information to the legitimate receiver. In this case, eavesdroppers distribute beyond the sector secrecy guard zone to intercept confidential information. Therefore, the SNR at the receiver is defined as

(5) $$\gamma_{D_{A}}=\frac{PM_{S}{\left|h_{D}\right|}^{2}L\left(r_{D}\right)}{\sigma_{D}^{2}},$$

and the instantaneous SNR of detecting the information of the legitimate receiver at the most detrimental eavesdropper is given by

(6) $$\gamma_{E_{A}}=\frac{\max_{E\in \Phi_{1}}\left(PM_{S}{\left|h_{E}\right|}^{2}L\left(r_{E_{A}}\right)\right)}{\sigma_{E}^{2}},$$

or

(7) $$\gamma_{E_{A1}}=\frac{\max_{E\in \Phi_{2}}\left(Pm_{S}{\left|h_{E}\right|}^{2}L\left(r_{E_{A1}}\right)\right)}{\sigma_{E}^{2}}.$$

where $E\in \Phi_{1}$ denotes that eavesdroppers reside in the signal beam out of the sector secrecy guard zone, and then the SNR at the eavesdropper is $\gamma_{E_{A}}$. $E\in \Phi_{2}$ denotes that eavesdroppers may reside anywhere except in the signal beam where the sector secrecy guard zone is located, and then the SNR at the eavesdropper is $\gamma_{E_{A1}}$. The distance $r_{E_{A}}$ from the eavesdropper to the transmitter is larger than the radius *r* of the sector secrecy guard zone. $r_{E_{A1}}$ is the distance from the eavesdropper to the transmitter in the side lobes, and $\sigma_{\nu }^{2},\nu \in \left\{D,E\right\}$ denotes the additive white Gaussian power.

### 3.2. Eavesdropper Is Detected in the Sector Secrecy Guard Zone

If there exist eavesdroppers within the sector secrecy guard zone, the transmitter emits AN with power $P_{A}$ while transmitting the signal with power $P_{S}$. The total transmit power is denoted as *P*, $P_{S}=\mu P,P_{A}=\left(1-\mu \right)P$, where μ is the power allocation factor of the confidential information power to the total transmit power *P* with 0≤μ≤1 [36]. Then, the SNR at the receiver is given by

(8) $$\gamma_{D_{B}}=\frac{P_{S}M_{S}{\left|h_{D}\right|}^{2}L\left(r_{D}\right)}{\sigma_{D}^{2}},$$

and the instantaneous SNR at the most detrimental eavesdropper is written as

(9) $$\gamma_{E_{B}}=\max_{E\in \Phi_{3}}\left\{\frac{\left(P_{S}M_{S}{\left|h_{E}\right|}^{2}L\left(r_{E_{B}}\right)\right)}{P_{A}M_{s}{\left|h_{E}\right|}^{2}L\left(r_{E_{B}}\right)+\sigma_{E}^{2}}\right\},$$

or

(10) $$\gamma_{E_{B1}}=\frac{\max_{E\in \Phi_{2}}\left(Pm_{S}{\left|h_{E}\right|}^{2}L\left(r_{E_{B1}}\right)\right)}{\sigma_{E}^{2}},$$

where $E\in \Phi_{3}$ denotes that eavesdroppers may reside in the sector secrecy guard zone, and then the SNR at the eavesdropper is $\gamma_{E_{B}}$. $\gamma_{E_{B1}}$ is the SNR of the eavesdropper at $E\in \Phi_{2}$. The distance $r_{E_{B}}$ from the eavesdropper to the transmitter is smaller than the radius *r* of the sector secrecy guard zone. $r_{E_{B1}}$ is the distance from the eavesdropper to the transmitter in the side lobes. Note that, in our paper, to prevent eavesdroppers from eavesdropping, the transmitter adds AN to the transmit signals. AN is generated so as to be canceled out at the legitimate receiver; thus, only eavesdroppers are affected by AN. Similar methods are presented in [39,40,41]. However, it is beyond the scope of our paper and could be our future research.

## 4. Performance Analysis

Hereinafter, we first analyze secrecy performance of the direct transmission in term of the COP, the SOP and the secrecy throughput. Then, according to the same performance metrics, AN assisted transmission is investigated. Actually, direct transmission and AN assisted transmission are two special cases of adaptive transmission. Finally, considering the probabilities aforementioned that eavesdroppers may be beyond the sector secrecy guard zone and within the sector secrecy guard zone, we study the secrecy performance of the adaptive transmission scheme.

### 4.1. Direct Transmission

For the direct transmission, there does not exist an eavesdropper within the sector secrecy guard zone, the transmitter keeps sending the confidential information to the legitimate receiver.

Substituting Equation (5) into Equation (3), the COP is derived as

(11) $$\begin{array}{cc} p_{coa} & =\;\;\;\mathrm{Pr}\;\;\left(\gamma_{D_{A}}\;\;<\;\;2^{R_{B}}\;-1\right)\;\;\;=\;\;\mathrm{Pr}\;\;\left(\;\;\frac{PM_{S}{\left|h_{D}\right|}^{2}L\left(r_{D}\right)}{\sigma_{D}^{2}}\;<\;2^{R_{B}}\;-\;1\right)=\mathrm{Pr}\left({\left|h_{D}\right|}^{2}<\frac{\left(2^{R_{B}}-1\right)\sigma_{D}^{2}}{PM_{S}L\left(r_{D}\right)}\right)\\ & =\sum_{i\in \left\{L,N\right\}}\left(\frac{\Upsilon \left(N_{i},\frac{\left(2^{R_{B}}-1\right)\sigma_{D}^{2}}{PM_{S}L\left(r_{D}\right)}N_{i}\right)}{\Gamma \left(N_{i}\right)}\right)P_{i}\left(r_{D}\right), \end{array}$$

where $\Upsilon \left(\cdot ,\cdot \right)$ is the lower incomplete gamma function [42] (Equation (8.350)).

In the case of $E\in \Phi_{1}$, substituting Equation (6) into Equation (2), the SOP is calculated as follows:

(12) $$p_{soa}=\mathrm{Pr}\left(\gamma_{E_{A}}>2^{R_{B}-R_{S}}-1\right)=\int_{2^{R_{B}-R_{S}}-1}^{\infty }f_{\gamma_{E_{A}}}\left(x\right)dx,$$

where $f_{\gamma_{E_{A}}}\left(\cdot \right)$ stands for the probability density function of $\gamma_{E_{A}}$. By using the thinning theorem [26,43,44], the eavesdroppers are divided into two independent PPPs, namely LOS point process $\Phi_{1}^{LOS}$ with density function $\lambda_{E}P_{L}\left(r_{d}\right)$, and NLOS point process $\Phi_{1}^{\mathrm{NOS}}$ with density function $\lambda_{E}\left(1-P_{L}\left(r_{d}\right)\right)$. Then, the cumulative distribution function of $\gamma_{E_{A}}$ is derived as

(13) $$\begin{array}{cc} F_{\gamma_{E_{A}}}\;\left(x\right) & \;\;\;=\;\;\;\mathrm{Pr}\;\left(\gamma_{E_{A}}\;<\;x\right)\;\;=\;\;\;\mathrm{Pr}\left\{\;\;\frac{\max_{E\in \Phi_{1}}\left(PM_{S}{\left|h_{E}\right|}^{2}L\left(r_{E_{A}}\right)\right)}{\sigma_{E}^{2}}\;\;<\;x\right\}\\ & \;=\underset{Z_{1}}{\underset{︸}{\mathrm{Pr}\left\{\frac{\max_{E\in \Phi_{1}^{L}}\left(PM_{S}{\left|h_{E}\right|}^{2}L\left(r_{E_{A}}\right)\right)}{\sigma_{E}^{2}}<x\right\}}}\times \underset{Z_{2}}{\underset{︸}{\mathrm{Pr}\left\{\frac{\max_{E\in \Phi_{1}^{N}}\left(PM_{S}{\left|h_{E}\right|}^{2}L\left(r_{E_{A}}\right)\right)}{\sigma_{E}^{2}}<x\right\}}}, \end{array}$$

where $\Phi_{1}^{L}$ and $\Phi_{1}^{N}$ are the set of LOS and NLOS eaversdroppers, respectively. $Z_{1}$ and $Z_{2}$ are calculated by Equations (14) and (15). In $Z_{1}$, step (a) follows the probability generating functional of the PPP, and step (b) is based on [42] (Equation (8.354.1)). In $Z_{2}$, step (c) follows the probability generating functional of the PPP, step (d) is based on [42] (Equation (3.381.9)):

(14) $$\begin{array}{c} \begin{array}{cc} Z_{1} & =\mathrm{Pr}\left\{\frac{\max_{E\in \Phi_{1}^{L}}\left(PM_{S}{\left|h_{E}\right|}^{2}L\left(r_{E}\right)\right)}{\sigma_{E}^{2}}<x\right\}\overset{a}{=}\exp\left(-\theta_{S}\lambda_{E}\int_{r}^{\infty }\mathrm{Pr}\left({\left|h_{E}\right|}^{2}>\frac{x\sigma_{E}^{2}r_{E}^{\alpha_{L}}}{PM_{S}C_{L}}\right)e^{-\beta r_{{}_{E}}}r_{E}dr_{E}\right)\\ & \overset{b}{=}\exp\left(-\theta_{S}\lambda_{E}\left(\frac{\Gamma \left(2,\beta r\right)}{\beta^{2}}-\frac{{\left(\frac{N_{L}x\sigma_{E}^{2}}{PM_{S}C_{L}}\right)}^{N_{L}}}{\Gamma \left(N_{L}\right)}\sum_{n=0}^{\infty }\frac{{\left(-1\right)}^{n}{\left(\frac{N_{L}x\sigma_{E}^{2}}{PM_{S}C_{L}}\right)}^{n}}{n!\left(N_{L}+n\right)}\frac{\Gamma \left(\alpha_{L}\left(N_{L}+n\right)+2,\beta r\right)}{\beta^{\alpha_{L}\left(N_{L}+n\right)+2}}\right)\right), \end{array} \end{array}$$

(15) $$\begin{array}{cc} Z_{2} & =\mathrm{Pr}\left\{\frac{\max_{E\in \Phi_{1}^{N}}\left(PM_{S}{\left|h_{E}\right|}^{2}L\left(r_{E_{A}}\right)\right)}{\sigma_{E}^{2}}<x\right\}=E\left\{\prod_{E\in \Phi_{1}^{N}}\mathrm{Pr}\left({\left|h_{E}\right|}^{2}<\frac{x\sigma_{E}^{2}}{PM_{S}L\left(r_{E_{A}}\right)}\right)|\Phi_{1}^{N}\right\}\\ & \overset{c}{=}\exp\left(-\theta_{S}\lambda_{E}\int_{r}^{\infty }\mathrm{Pr}\left({\left|h_{E}\right|}^{2}>\frac{x\sigma_{E}^{2}r_{E_{A}}^{\alpha_{N}}}{PM_{S}C_{N}}\right)\left(1-e^{-\beta r_{{}_{E_{A}}}}\right)r_{E_{A}}dr_{E_{A}}\right)\\ & \overset{d}{=}\exp\left(-\theta_{S}\lambda_{E}\left(\left(\frac{\left(N_{N}-1\right)!}{\Gamma \left(N_{N}\right)}\right)\sum_{m=0}^{N_{N}-1}\frac{{\left(\frac{x\sigma_{E}^{2}N_{N}}{PM_{S}C_{N}}\right)}^{m}}{m!}\times \frac{\Gamma \left(\frac{m\alpha_{N}+2}{\alpha_{N}},\frac{x\sigma_{E}^{2}N_{N}}{PM_{S}C_{N}}r^{\alpha_{N}}\right)}{\alpha_{N}{\left(\frac{x\sigma_{E}^{2}N_{N}}{PM_{S}C_{N}}\right)}^{\frac{m\alpha_{N}+2}{\alpha_{N}}}}\right.\right.\\ & \left.\left.-\left(\frac{\Gamma \left(2,\beta r\right)}{\beta^{2}}-\frac{{\left(\frac{N_{N}x\sigma_{E}^{2}}{PM_{S}C_{N}}\right)}^{N_{N}}}{\Gamma \left(N_{N}\right)}\sum_{n=0}^{\infty }\frac{{\left(-1\right)}^{n}{\left(\frac{N_{N}x\sigma_{E}^{2}}{PM_{S}C_{N}}\right)}^{n}}{n!\left(N_{N}+n\right)}\frac{\Gamma \left(\alpha_{N}\left(N_{N}+n\right)+2,\beta r\right)}{\beta^{\alpha_{N}\left(N_{N}+n\right)+2}}\right)\right)\right). \end{array}$$

For the sake of simplicity, $F_{\gamma_{E_{A}}}\left(x\right)$ can be simplified as

(16) $$F_{\gamma_{E_{A}}}\left(x\right)=\exp\left(-\theta_{S}\lambda_{E}\left(B+A_{N}-A_{L}\right)\right),$$

where $B=\left(\frac{\left(N_{N}-1\right)!}{\Gamma \left(N_{N}\right)}\right)\sum_{m=0}^{N_{N}-1}\frac{q^{m}}{m!}\times \frac{\Gamma \left(\frac{m\alpha_{N}+2}{\alpha_{N}},qr^{\alpha_{N}}\right)}{\alpha_{N}q^{\frac{m\alpha_{N}+2}{\alpha_{N}}}}$; here, $\Gamma \left(\cdot ,\cdot \right)$ is the upper incomplete gamma function [42] (Equation (8.350)), $A_{N}=\frac{q^{N_{N}}}{\Gamma \left(N_{N}\right)}\sum_{n=0}^{\infty }\frac{{\left(-1\right)}^{n}q^{n}}{n!\left(N_{N}+n\right)}\frac{\Gamma \left(\alpha_{N}\left(N_{N}+n\right)+2,\beta r\right)}{\beta^{\alpha_{N}\left(N_{N}+n\right)+2}}$, $A_{L}=\frac{p^{N_{L}}}{\Gamma \left(N_{L}\right)}\sum_{n=0}^{\infty }\frac{{\left(-1\right)}^{n}p^{n}}{n!\left(N_{L}+n\right)}\frac{\Gamma \left(\alpha_{L}\left(N_{L}+n\right)+2,\beta r\right)}{\beta^{\alpha_{L}\left(N_{L}+n\right)+2}}$, $q=\frac{x\sigma_{E}^{2}N_{N}}{PM_{S}C_{N}}$, $p=\frac{N_{L}x\sigma_{E}^{2}}{PM_{S}C_{L}}$.

Based on Equations (12) and (16), the SOP is derived as

(17) $$\begin{array}{cc} p_{soa} & =\mathrm{Pr}\left(\gamma_{E_{A}}>2^{R_{B}-R_{S}}-1\right)=\int_{2^{R_{B}-R_{S}}-1}^{\infty }f_{\gamma_{E_{A}}}\left(x\right)dx\\ & =1-\exp\left(-\theta_{S}\lambda_{E}\left(B+A_{N}-A_{L}\right)\right). \end{array}$$

Substituting Equations (11) and (17) into Equation (4), the secrecy throughput $\eta_{a}$ is derived as

(18) $$\begin{array}{cc} \eta_{a} & =\left(1-p_{coa}\right)\left(1-p_{soa}\right)R_{S}\\ & =\left(1-\sum_{i\in \left\{L,N\right\}}\left(\frac{\Upsilon \left(N_{i},\frac{\left(2^{R_{B}}-1\right)\sigma_{D}^{2}}{PM_{S}L\left(r_{D}\right)}N_{i}\right)}{\Gamma \left(N_{i}\right)}\right)P_{i}\left(r_{D}\right)\right)\\ & \times \exp\left(-\theta_{S}\lambda_{E}\left(B+A_{N}-A_{L}\right)\right)R_{S}. \end{array}$$

**Remark** **1.** *It can be deduced from Equation (11) that the COP is a decreasing function of P, this implies that the reliability performance of system is strengthened as P increases. For the case of $E\in \Phi_{1}$, Equation (17) shows that the SOP increases with P when the sector secrecy guard zone is invariant. Correspondingly, when P is fixed, the secrecy performance becomes better with the increase of the sector secrecy guard zone. It means that, in this case, increasing power makes it possible to leak confidential information to eavesdroppers, and, at the same time, using the sector guard zone to keep eavesdroppers away from legitimate user. In addition, from Equation (18), it can be deduced that, in addition to P and $\lambda_{E}$, the secrecy throughput has a close relationship with radius r and central angle $\theta_{S}$ of the secrecy guard zone.*

In the case of $E\in \Phi_{2}$, i.e., eavesdroppers may reside anywhere except in the signal beam where the sector secrecy guard zone is located. Substituting Equation (7) into Equation (2), the SOP is derived as

(19) $$p_{soa1}=\mathrm{Pr}\left(\gamma_{E_{A1}}>2^{R_{B}-R_{S}}-1\right)=\int_{2^{R_{B}-R_{S}}-1}^{\infty }f_{\gamma_{E_{A1}}}\;\;\left(x\right)dx.$$

The cumulative distribution function of $\gamma_{E_{A1}}$ is derived as

(20) $$\begin{array}{cc} F_{\gamma_{E_{A1}}}\;\;\left(x\right)\;\;\; & \;=\;\;\;\mathrm{Pr}\;\;\left(\;\gamma_{E_{A1}}\;\;\;<x\right)\;\;\;=\;\;\;\mathrm{Pr}\;\left\{\;\;\frac{\max_{E\in \Phi_{2}}\left(Pm_{S}{\left|h_{E}\right|}^{2}L\left(r_{E_{A1}}\right)\right)}{\sigma_{E}^{2}}\;\;<x\right\}\\ & =\underset{Z_{3}}{\underset{︸}{\mathrm{Pr}\left\{\frac{\max_{E\in \Phi_{2}^{L}}\left(Pm_{S}{\left|h_{E}\right|}^{2}L\left(r_{E_{A1}}\right)\right)}{\sigma_{E}^{2}}<x\right\}}}\times \underset{Z_{4}}{\underset{︸}{\mathrm{Pr}\left\{\frac{\max_{E\in \Phi_{2}^{N}}\left(Pm_{S}{\left|h_{E}\right|}^{2}L\left(r_{E_{A1}}\right)\right)}{\sigma_{E}^{2}}<x\right\}}}, \end{array}$$

where $\Phi_{2}^{L}$ and $\Phi_{2}^{N}$ are the set of LOS and NLOS eaversdroppers, respectively. $Z_{3}$ and $Z_{4}$ are calculated by (14) and (15). In $Z_{3}$, step (e) is based on [42] (Equation (3.351.2)). In $Z_{4}$, step (f) is based on [42] (Equation (3.381.9)):

(21) $$\begin{array}{cc} Z_{3} & =\mathrm{Pr}\left\{\frac{\max_{E\in \Phi_{2}^{L}}\left(Pm_{S}{\left|h_{E}\right|}^{2}L\left(r_{E_{A1}}\right)\right)}{\sigma_{E}^{2}}<x\right\}=E\left\{\prod_{E\in \Phi_{2}^{L}}\mathrm{Pr}\left({\left|h_{E}\right|}^{2}<\frac{x\sigma_{E}^{2}}{Pm_{S}L\left(r_{E_{A1}}\right)}\right)|\Phi_{2}^{L}\right\}\\ & \overset{e}{=}\exp\left(-\left(2\pi -\theta_{S}\right)\lambda_{E}\left(\frac{\Gamma \left(2\right)}{\beta^{2}}-\frac{{\left(\frac{N_{L}x\sigma_{E}^{2}}{Pm_{S}C_{L}}\right)}^{N_{L}}}{\Gamma \left(N_{L}\right)}\sum_{n=0}^{\infty }\frac{{\left(-1\right)}^{n}{\left(\frac{N_{L}x\sigma_{E}^{2}}{Pm_{S}C_{L}}\right)}^{n}}{n!\left(N_{L}+n\right)}\frac{\Gamma \left(\alpha_{L}\left(N_{L}+n\right)+2\right)}{\beta^{\alpha_{L}\left(N_{L}+n\right)+2}}\right)\right), \end{array}$$

(22) $$\begin{array}{cc} Z_{4} & =\mathrm{Pr}\left\{\frac{\max_{E\in \Phi_{2}^{N}}\left(Pm_{S}{\left|h_{E}\right|}^{2}L\left(r_{E_{A1}}\right)\right)}{\sigma_{E}^{2}}<x\right\}=E\left\{\prod_{E\in \Phi_{2}^{N}}\mathrm{Pr}\left({\left|h_{E}\right|}^{2}<\frac{x\sigma_{E}^{2}}{Pm_{S}L\left(r_{E_{A1}}\right)}\right)|\Phi_{2}^{N}\right\}\\ & \overset{f}{=}\exp\left(-\left(2\pi -\theta_{S}\right)\lambda_{E}\left(\left(\frac{\left(N_{N}-1\right)!}{\Gamma \left(N_{N}\right)}\right)\sum_{m=0}^{N_{N}-1}\frac{{\left(\frac{x\sigma_{E}^{2}N_{N}}{Pm_{S}C_{N}}\right)}^{m}}{m!}\times \frac{\Gamma \left(\frac{m\alpha_{N}+2}{\alpha_{N}}\right)}{\alpha_{N}{\left(\frac{x\sigma_{E}^{2}N_{N}}{Pm_{S}C_{N}}\right)}^{\frac{m\alpha_{N}+2}{\alpha_{N}}}}\right.\right.\\ & \left.\left.-\left(\frac{\Gamma \left(2\right)}{\beta^{2}}-\frac{{\left(\frac{N_{N}x\sigma_{E}^{2}}{Pm_{S}C_{N}}\right)}^{N_{N}}}{\Gamma \left(N_{N}\right)}\sum_{n=0}^{\infty }\frac{{\left(-1\right)}^{n}{\left(\frac{N_{N}x\sigma_{E}^{2}}{Pm_{S}C_{N}}\right)}^{n}}{n!\left(N_{N}+n\right)}\frac{\Gamma \left(\alpha_{N}\left(N_{N}+n\right)+2\right)}{\beta^{\alpha_{N}\left(N_{N}+n\right)+2}}\right)\right)\right). \end{array}$$

Upon further simplification, $F_{\gamma_{E_{A1}}}\left(x\right)$ can be simplified as

(23) $$F_{\gamma_{E_{A1}}}\left(x\right)=\exp\left(-\left(2\pi -\theta_{S}\right)\lambda_{E}\left(D+C_{N}-C_{L}\right)\right),$$

where $D=\left(\frac{\left(N_{N}-1\right)!}{\Gamma \left(N_{N}\right)}\right)\sum_{m=0}^{N_{N}-1}\frac{u^{m}}{m!}\times \frac{\Gamma \left(\frac{m\alpha_{N}+2}{\alpha_{N}}\right)}{\alpha_{N}u^{\frac{m\alpha_{N}+2}{\alpha_{N}}}}$, $u=\frac{x\sigma_{E}^{2}N_{N}}{Pm_{S}C_{N}}$, $C_{N}=\frac{u^{N_{N}}}{\Gamma \left(N_{N}\right)}\sum_{n=0}^{\infty }\frac{{\left(-1\right)}^{n}u^{n}}{n!\left(N_{N}+n\right)}\frac{\Gamma \left(\alpha_{N}\left(N_{N}+n\right)+2\right)}{\beta^{\alpha_{N}\left(N_{N}+n\right)+2}}$, $C_{L}\;=\;\frac{k^{N_{L}}}{\Gamma \left(N_{L}\right)}\sum_{n=0}^{\infty }\frac{{\left(-1\right)}^{n}k^{n}}{n!\left(N_{L}+n\right)}\frac{\Gamma \left(\alpha_{L}\left(N_{L}+n\right)+2\right)}{\beta^{\alpha_{L}\left(N_{L}+n\right)+2}}$, $k=\frac{N_{L}x\sigma_{E}^{2}}{Pm_{S}C_{L}}$.

Based on Equations (19) and (23), the SOP is derived as

(24) $$\begin{array}{cc} p_{soa1} & =\mathrm{Pr}\left(\gamma_{E_{A1}}>2^{R_{B}-R_{S}}-1\right)=\int_{2^{R_{B}-R_{S}}-1}^{\infty }f_{\gamma_{E_{A1}}}\left(x\right)dx\\ & =1-\exp\left(-\left(2\pi -\theta_{S}\right)\lambda_{E}\left(D+C_{N}-C_{L}\right)\right). \end{array}$$

Substituting Equations (11) and (24) into Equation (4), the secrecy throughput is derived as

(25) $$\begin{array}{cc} \eta_{soa1} & =\left(1-p_{coa}\right)\left(1-p_{soa1}\right)R_{S}\\ & =\left(1-\sum_{i\in \left\{L,N\right\}}\left(\frac{\Upsilon \left(N_{i},\frac{\left(2^{R_{B}}-1\right)\sigma_{D}^{2}}{PM_{S}L\left(r_{D}\right)}N_{i}\right)}{\Gamma \left(N_{i}\right)}\right)P_{i}\left(r_{D}\right)\right)\\ & \times \exp\left(-\left(2\pi -\theta_{S}\right)\lambda_{E}\left(D+C_{N}-C_{L}\right)\right)R_{S}. \end{array}$$

**Remark** **2.** *In the case of $E\in \Phi_{2}$, the eavesdroppers are not in the beam where the sector secrecy guard zone is located; security threats come mainly from sidelobe. Referecne Equation (24) shows that the SOP is an increasing function of P and $m_{S}$, which indicates that the secrecy performance becomes better as P and $m_{S}$ decreases. It means that, when the eavesdroppers are in the sidelobe, increasing power P and $m_{S}$ may lead to a leakage of confidential information. At the same time, increasing the central angle $\theta_{S}$ of the sector guard zone can reduce the risk of eavesdropping. In addition, from Equation (25), it can be deduced that, in addition to P and eavesdropper density $\lambda_{E}$, the secrecy throughput has a close relationship with radius r and central angle $\theta_{S}$ of the secrecy guard zone as well.*

On the basis of the locations for the eavesdroppers, both $E\in \Phi_{1}$ and $E\in \Phi_{2}$ are considered together, i.e., eavesdroppers may reside anywhere except the sector secrecy guard zone. The SOP is derived as

(26) $$\begin{array}{cc} p_{sot} & =\mathrm{Pr}\left(\max\left(\gamma_{E_{A}},\gamma_{E_{A1}}\right)>2^{R_{B}-R_{S}}-1\right)\\ & =1\;\;-\;\;\mathrm{Pr}\;\;\left(\gamma_{E_{A}}\;<\;2^{R_{B}-R_{S}}\;-\;1\right)\times \mathrm{Pr}\left(\gamma_{E_{A1}}\;<\;2^{R_{B}-R_{S}}\;-\;1\right)\\ & =\;1-\exp\left(-\theta_{S}\lambda_{E}\left(B+A_{N}-A_{L}\right)\right.\left.-\left(2\pi -\theta_{S}\right)\lambda_{E}\left(D+C_{N}-C_{L}\right)\right). \end{array}$$

The total secrecy throughput of eavesdroppers in $E\in \Phi_{1}$ and $E\in \Phi_{2}$ is derived as

(27) $$\begin{array}{cc} \eta_{t} & =\left(1-p_{coa}\right)\left(1-p_{sot}\right)R_{S}\\ & =\left(1-\sum_{i\in \left\{L,N\right\}}\left(\frac{\Upsilon \left(N_{i},\frac{\left(2^{R_{B}}-1\right)\sigma_{D}^{2}}{PM_{S}L\left(r_{D}\right)}N_{i}\right)}{\Gamma \left(N_{i}\right)}\right)P_{i}\left(r_{D}\right)\right)\\ & \times \exp\left(-\theta_{S}\lambda_{E}\left(B+A_{N}-A_{L}\right)\right.\left.-\left(2\pi -\theta_{S}\right)\lambda_{E}\left(D+C_{N}-C_{L}\right)\right)R_{S}. \end{array}$$

### 4.2. AN Assisted Transmission

For AN assisted transmission, once the eavesdroppers locate in the sector secrecy guard zone, the transmitter emits information-bearing signal along with AN.

Thus, substituting Equation (8) into Equation (3), the COP is derived as

(28) $$\begin{array}{cc} p_{cob}\;\; & \;=P\left(\;\gamma_{D_{B}}\;\;<\;\;2^{R_{B}}\;-\;1\right)\;\;=\;\mathrm{Pr}\left(\;\frac{P_{S}M_{S}{\left|h_{D}\right|}^{2}L\left(r_{D}\right)}{\sigma_{D}^{2}}\;<\;2^{R_{B}}\;-\;1\right)\\ & \;=\;\sum_{i\in \left\{L,N\right\}}\mathrm{Pr}\left(\left.{\left|h_{D}\right|}^{2}<\frac{\left(2^{R_{B}}-1\right)\sigma_{D}^{2}}{P_{S}M_{S}L\left(r_{D}\right)}\right|i\right)P_{i}\left(r_{D}\right)\\ & \;=\;\sum_{i\in \left\{L,N\right\}}\left(\frac{\Upsilon \left(N_{i},\frac{\left(2^{R_{B}}-1\right)\sigma_{D}^{2}}{P_{S}M_{S}L\left(r_{D}\right)}N_{i}\right)}{\Gamma \left(N_{i}\right)}\right)P_{i}\left(r_{D}\right). \end{array}$$

In the case of $E\in \Phi_{3}$, i.e., eavesdroppers reside in the sector secrecy guard zone. Substituting Equation (9) into Equation (2), the SOP is derived as

(29) $$p_{sob}=\mathrm{Pr}\left(\gamma_{E_{B}}>2^{R_{B}-R_{S}}-1\right)=\int_{2^{R_{B}-R_{S}}-1}^{\infty }f_{\gamma_{E_{B}}}\left(x\right)dx,$$

where $f_{\gamma_{E_{B}}}\left(\cdot \right)$ stands for the probability density function of $\gamma_{E_{B}}$; then, the cumulative distribution function of $\gamma_{E_{B}}$ is derived as

(30) $$\begin{array}{c} F_{\gamma_{E_{B}}}\left(x\right)=\mathrm{Pr}\left(\gamma_{E_{B}}<x\right)\\ =\mathrm{Pr}\left\{\max_{E\in \Phi_{3}}\left\{\frac{\left(P_{S}M_{S}{\left|h_{E}\right|}^{2}L\left(r_{E_{B}}\right)\right)}{P_{A}M_{s}{\left|h_{E}\right|}^{2}L\left(r_{E}\right)+\sigma_{E}^{2}}\right\}<x\right\}\\ =\underset{Z_{5}}{\underset{︸}{\mathrm{Pr}\left\{\max_{E\in \Phi_{{}_{3}}^{L}}\left\{\frac{\left(P_{S}M_{S}{\left|h_{E}\right|}^{2}L\left(r_{E_{B}}\right)\right)}{P_{A}M_{s}{\left|h_{E}\right|}^{2}L\left(r_{E_{B}}\right)+\sigma_{E}^{2}}\right\}<x\right\}}}\times \underset{Z_{6}}{\underset{︸}{\mathrm{Pr}\left\{\max_{E\in \Phi_{{}_{3}}^{N}}\left\{\frac{\left(P_{S}M_{S}{\left|h_{E}\right|}^{2}L\left(r_{E_{B}}\right)\right)}{P_{A}M_{s}{\left|h_{E}\right|}^{2}L\left(r_{E_{B}}\right)+\sigma_{E}^{2}}\right\}<x\right\}}}, \end{array}$$

where $\Phi_{3}^{L}$ and $\Phi_{3}^{N}$ are the set of LOS and NLOS eavesdroppers, respectively. $Z_{5}$ and $Z_{6}$ are calculated by Equations (31) and (32). In $Z_{5}$, step (g) follows the probability generating functional of the PPP, step (h) is based on [42] (Equation(3.351.2)). In $Z_{6}$, step (p) follows the probability generating functional of the PPP, step (q) is based on [42] (Equation (3.381.9)):

(31) $$\begin{array}{cc} Z_{5} & =\mathrm{Pr}\left\{\max_{E\in \Phi_{{}_{3}}^{L}}\left\{\frac{\left(P_{S}M_{S}{\left|h_{E}\right|}^{2}L\left(r_{E_{B}}\right)\right)}{P_{A}M_{s}{\left|h_{E}\right|}^{2}L\left(r_{E_{B}}\right)+\sigma_{E}^{2}}\right\}<x\right\}\\ & \;\overset{g}{=}U\left(x-\frac{P_{S}}{P_{A}}\right)+U\left(\frac{P_{S}}{P_{A}}-x\right)\exp\left(-\theta_{S}\lambda_{E}\int_{0}^{r}\mathrm{Pr}\left({\left|h_{E}\right|}^{2}>\frac{x\sigma_{E}^{2}}{L\left(r_{E_{B}}\right)\left(P_{S}M_{S}-P_{A}M_{S}x\right)}\right)e^{-\beta r_{E_{B}}}r_{E_{B}}dr_{E_{B}}\right)\\ & \;\overset{h}{=}U\left(x-\frac{P_{S}}{P_{A}}\right)+U\left(\frac{P_{S}}{P_{A}}-x\right)\exp\left(-\theta_{S}\lambda_{E}\left(\frac{\Upsilon \left(2,\beta r\right)}{\beta^{2}}-\frac{v^{N_{L}}}{\Gamma \left(N_{L}\right)}\sum_{n=0}^{\infty }\frac{{\left(-1\right)}^{n}v^{n}}{n!\left(N_{L}+n\right)}\frac{\Upsilon \left(\alpha_{L}\left(N_{L}+n\right)+2,\beta r\right)}{\beta^{\alpha_{L}\left(N_{L}+n\right)+2}}\right)\right), \end{array}$$

(32) $$\begin{array}{cc} Z_{6} & =\mathrm{Pr}\left\{\max_{E\in \Phi_{3}^{N}}\left\{\frac{\left(P_{S}M_{S}{\left|h_{E}\right|}^{2}L\left(r_{E_{B}}\right)\right)}{P_{A}M_{s}{\left|h_{E}\right|}^{2}L\left(r_{E_{B}}\right)+\sigma_{E}^{2}}\right\}<x\right\}\\ & \overset{p}{=}U\left(x\;-\;\frac{P_{S}}{P_{A}}\right)+U\left(\frac{P_{S}}{P_{A}}\;-\;x\right)\exp\left(\;-\;\;\theta_{S}\lambda_{E}\int_{0}^{r}\mathrm{Pr}\left({\left|h_{E}\right|}^{2}>\frac{x\sigma_{E}^{2}}{L\left(r_{E_{B}}\right)\left(P_{S}M_{S}-P_{A}M_{S}x\right)}\right)\left(1\;-\;e^{\;-\;\beta r_{E_{B}}}\right)r_{E_{B}}dr_{E_{B}}\right)\\ & \overset{q}{=}U\left(x-\frac{P_{S}}{P_{A}}\right)+U\left(\frac{P_{S}}{P_{A}}-x\right)\exp\left(-\theta_{S}\lambda_{E}\left(\left(\frac{\left(N_{N}-1\right)!}{\Gamma \left(N_{N}\right)}\right)\sum_{m=0}^{N_{N}-1}\frac{w^{m}}{m!}\times \frac{\Upsilon \left(\frac{m\alpha_{N}+2}{\alpha_{N}},wr^{\alpha_{N}}\right)}{\alpha_{N}w^{\frac{m\alpha_{N}+2}{\alpha_{N}}}}\right.\right.\\ & \left.\left.\;\;\;\;-\left(\frac{\Upsilon \left(2,\beta r\right)}{\beta^{2}}-\frac{w^{N_{N}}}{\Gamma \left(N_{N}\right)}\sum_{n=0}^{\infty }w\frac{\Upsilon \left(\alpha_{N}\left(N_{N}+n\right)+2,\beta r\right)}{\beta^{\alpha_{N}\left(N_{N}+n\right)+2}}\right)\right)\right). \end{array}$$

Upon further simplification, $F_{\gamma_{E_{B}}}\left(x\right)$ can be simplified as

(33) $$F_{\gamma_{E_{B}}}\left(x\right)=\exp\left(-\theta_{S}\lambda_{E}\left(F+E_{N}-E_{L}\right)\right),$$

where $F=\left(\frac{\left(N_{N}-1\right)!}{\Gamma \left(N_{N}\right)}\right)\sum_{m=0}^{N_{N}-1}\frac{w^{m}}{m!}\times \frac{\Upsilon \left(\frac{m\alpha_{N}+2}{\alpha_{N}},wr^{\alpha_{N}}\right)}{\alpha_{N}w^{\frac{m\alpha_{N}+2}{\alpha_{N}}}}$, $E_{N}=\frac{w^{N_{N}}}{\Gamma \left(N_{N}\right)}\sum_{n=0}^{\infty }\frac{{\left(-1\right)}^{n}w^{n}}{n!\left(N_{N}+n\right)}\times \frac{\Upsilon \left(\alpha_{N}\left(N_{N}+n\right)+2,\beta r\right)}{\beta^{\alpha_{N}\left(N_{N}+n\right)+2}}$, and $w=\frac{x\sigma_{E}^{2}N_{N}}{\left(P_{S}M_{S}-P_{A}M_{S}x\right)C_{N}}$, $v=\frac{N_{L}x\sigma_{E}^{2}}{\left(P_{S}M_{S}-P_{A}M_{S}x\right)C_{L}}$, and $E_{L}=\frac{v^{N_{L}}}{\Gamma \left(N_{L}\right)}\sum_{n=0}^{\infty }\frac{{\left(-1\right)}^{n}v^{n}}{n!\left(N_{L}+n\right)}\frac{\Upsilon \left(\alpha_{L}\left(N_{L}+n\right)+2,\beta r\right)}{\beta^{\alpha_{L}\left(N_{L}+n\right)+2}}$.

Based on Equations (29) and (33), the SOP is derived as

(34) $$\begin{array}{cc} p_{sob} & =\mathrm{Pr}\left(\gamma_{E_{B}}>2^{R_{B}-R_{S}}-1\right)=\int_{2^{R_{B}-R_{S}}-1}^{\infty }f_{\gamma_{E_{B}}}\left(x\right)dx\\ & =1-\exp\left(-\theta_{S}\lambda_{E}\left(F+E_{N}-E_{L}\right)\right). \end{array}$$

Substituting Equations (28) and (34) into Equation (4), the secrecy throughput is derived as

(35) $$\begin{array}{cc} \eta_{b} & =\left(1-p_{cob}\right)\left(1-p_{sob}\right)R_{S}\\ & =\left(1-\sum_{i\in \left\{L,N\right\}}\left(\frac{\Upsilon \left(N_{i},\frac{\left(2^{R_{B}}-1\right)\sigma_{D}^{2}}{P_{S}M_{S}L\left(r_{D}\right)}N_{i}\right)}{\Gamma \left(N_{i}\right)}\right)P_{i}\left(r_{D}\right)\right)\\ & \times \exp\left(-\theta_{S}\lambda_{E}\left(F+E_{N}-E_{L}\right)\right)R_{S}. \end{array}$$

**Remark** **3.** *From Equation (28), it is explicitly shown that the COP is a decreasing function about transmitting power. Adding more transmitting power could help the improvement of reliability performance. In the case of $E\in \Phi_{3}$, the eavesdroppers are located in the sector secrecy guard zone, and the transmitter allocates a portion of the power to transmit the AN to confuse the eavesdroppers. From Equation (34), we see that the SOP has a close relationship with central angle $\theta_{S}$, eavesdropper density $\lambda_{E}$ and AN power. Additionally, Equation (35) shows that, in addition to central angle $\theta_{S}$ and eavesdropper density $\lambda_{E}$, the transmit power allocation factor μ is of vital importance.*

In addition, the eavesdroppers may be in the case of $E\in \Phi_{1}$ and $E\in \Phi_{2}$, the derivation process and results are similar to those in Section 4.1.

### 4.3. Adaptive Transmission

In order to adapt to the actual scenario, both $E\in \Phi_{1}$, $E\in \Phi_{2}$ and $E\in \Phi_{3}$ are considered together, and the adaptive transmission scheme is adopted. That is, when the eavesdroppers are beyond the sector secrecy guard zone, the system adopts direct transmission, and when the eavesdroppers are in the sector secrecy guard zone, AN assisted transmission is used. Considering the probabilities aforementioned that eavesdroppers may be beyond the sector secrecy guard zone and within the sector secrecy guard zone, we study the secrecy performance of an adaptive transmission scheme.

Combined with the probability $p_{e1}$ that the eavesdroppers may not be in sector secrecy guard zone, we deduce the SOP, which is derived as

(36) $$\begin{array}{cc} p_{soc1} & =p_{e1}\times \mathrm{Pr}\left(\max\left(\gamma_{E_{A}},\gamma_{E_{A1}}\right)>2^{R_{B}-R_{S}}-1\right)\\ & =p_{e1}\times \left(1-\exp\left(-\theta_{S}\lambda_{E}\left(B+A_{N}-A_{L}\right)\right.\right.\left.\left.-\left(2\pi -\theta_{S}\right)\lambda_{E}\left(D+C_{N}-C_{L}\right)\right)\right). \end{array}$$

Correspondingly, considering the probability $p_{e2}$ of eavesdroppers in sector secrecy guard zone, we deduce SOP, which is expressed as

(37) $$\begin{array}{cc} p_{soc2} & =p_{e2}\times \mathrm{Pr}\left(\max\left(\gamma_{E_{A1}},\gamma_{E_{B}}\right)>2^{R_{B}-R_{S}}-1\right)\\ & =p_{e2}\times \left(1-\exp\left(-\theta_{S}\lambda_{E}\left(F+E_{N}-E_{L}\right)\right.\right.\left.\left.-\left(2\pi -\theta_{S}\right)\lambda_{E}\left(D+C_{N}-C_{L}\right)\right)\right). \end{array}$$

Finally, we derive the SOP of the whole system under the random distribution of eavesdroppers, which is written as

(38) $$\begin{array}{cc} p_{soc} & =p_{soc1}+p_{soc2}\\ & =p_{e1}\times \left(1-\exp\left(-\theta_{S}\lambda_{E}\left(B+A_{N}-A_{L}\right)\right.\right.\left.\left.-\left(2\pi -\theta_{S}\right)\lambda_{E}\left(D+C_{N}-C_{L}\right)\right)\right)\\ & +p_{e2}\times \left(1-\exp\left(-\theta_{S}\lambda_{E}\left(F+E_{N}-E_{L}\right)\right.\right.\left.\left.-\left(2\pi -\theta_{S}\right)\lambda_{E}\left(D+C_{N}-C_{L}\right)\right)\right). \end{array}$$

As a result, the total secrecy throughput of the whole system is derived as

(39) $$\begin{array}{cc} \eta & =\left(1-p_{cob}\right)\left(1-p_{soc}\right)R_{S}\\ & =\left(1-\sum_{i\in \left\{L,N\right\}}\left(\frac{\Upsilon \left(N_{i},\frac{\left(2^{R_{B}}-1\right)\sigma_{D}^{2}}{PM_{S}L\left(r_{D}\right)}N_{i}\right)}{\Gamma \left(N_{i}\right)}\right)P_{i}\left(r_{D}\right)\right)\\ & \times \left(1-p_{e1}\times \left(1-\exp\left(-\theta_{S}\lambda_{E}\left(B+A_{N}-A_{L}\right)\right.\right.\right.\left.\left.-\left(2\pi -\theta_{S}\right)\lambda_{E}\left(D+C_{N}-C_{L}\right)\right)\right)\\ & \;-p_{e2}\times \left(1-\exp\left(-\theta_{S}\lambda_{E}\left(F+E_{N}-E_{L}\right)\right.\right.\left.\left.\left.-\left(2\pi -\theta_{S}\right)\lambda_{E}\left(D+C_{N}-C_{L}\right)\right)\right)\right)R_{S}. \end{array}$$

## 5. Numerical Results

In this section, some representative simulation results are presented to verify our theoretical analysis, and characterize the secrecy performance of the mmWave wiretap network. A set of closed-form expressions are derived in an adaptive transmission scheme to analyze the effects for different system parameters. We assume that the noise power is $\sigma_{D}^{2}=\sigma_{E}^{2}=-70$ dBm, and the LOS probability function is $P_{L}\left(r\right)=e^{-\beta r}$ with $\frac{1}{\beta }=141.4$. According to [45], we focus on the carrier frequenciey of 28 GHz and 73 GHz. The Nakagami fading parameters of the LOS (NLOS) link are $N_{L}=3$ ($N_{N}=2$), the parameters of path-loss model are $\beta_{L}=61.4\;\mathrm{dB},\;\alpha_{L}=2,\;\beta_{N}=72\;\mathrm{dB},\;\alpha_{N}=2.92$ and $\beta_{L}=69.8\;\mathrm{dB},\;\alpha_{L}=2,\;\beta_{N}=82.7\;\mathrm{dB},\;\alpha_{N}=2.69$, $C_{L}=10^{-\frac{\beta_{L}}{10}}$ and $C_{N}=10^{-\frac{\beta_{N}}{10}}$ can be regarded as path-loss intercepts on the reference distance of LOS and NLOS links.

**Figure 2:**
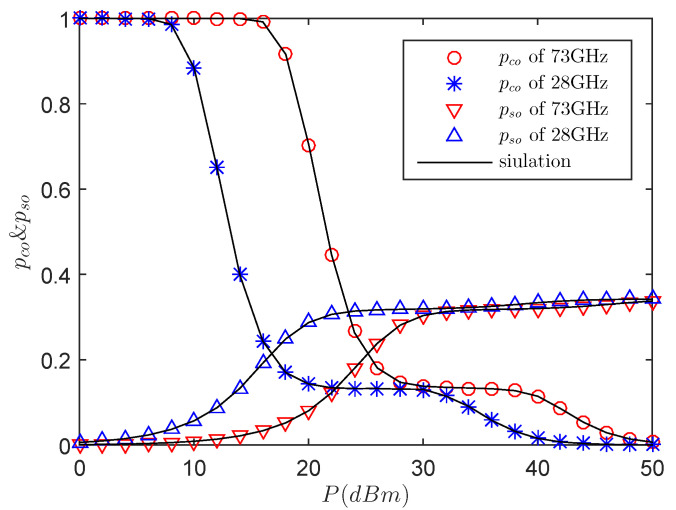
The $p_{co}$ and $p_{so}$ versus *P* with $R_{B}=1.5$ bps/Hz, $R_{S}=0.5$ bps/Hz, $\lambda_{E}=0.0002$ ${\mathrm{nodes}/m}^{2}$, r=20 m, $\theta_{S}=\frac{\pi }{3}$, μ = 0.6, $m_{S}=0.1$ and $M_{S}=200$.

Figure 2 presents the effects of the $p_{co}$ and $p_{so}$ versus the transmit power in different frequency bands, namely 28 GHz and 73 GHz. Obviously, with the increasing of *P*, the reliability performance of the legitimate receiver increases due to the decrease of the COP for a given power allocation factor, while the secrecy performance would decline. When the power increases to a certain value, the SNR received by the eavesdropper is close to a fixed value from Equation (6), the SOP remains unchanged and the COP of the legitimate receiver is close to zero. This can be explained as follows: on the one hand, although the eavesdropper is in the sector guard zone, the system can still guarantee a secure link to a legitimate receiver by transmitting AN to confuse the eavesdropper. On the other hand, it is because *P* has different effects on the COP and SOP in the case of LOS and NLOS. In addition, when *P* is large, the difference of SNR between legitimate link and eavesdropping link tends to be constant. It means that the reliability of the system can be improved effectively by increasing the power of the system. Again, we obtain an important observation that secrecy transmission at 28 GHz is better than that at 73 GHz in a low transmit power region.

**Figure 3:**
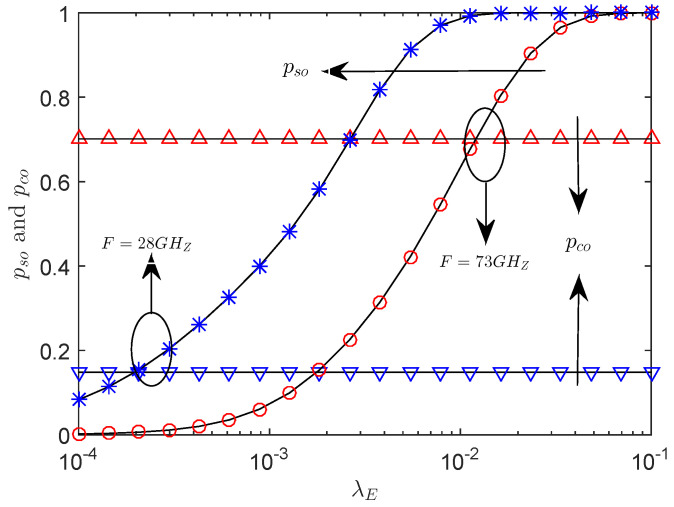
The $p_{co}$ and $p_{so}$ versus λ with $R_{B}=1.5$ bps/Hz, $R_{S}=0.5$ bps/Hz, P=30 dBm, r=50 m, $\theta_{S}=\frac{\pi }{3}$, μ = 0.6, $m_{S}=0.1$ and $M_{S}=200$.

Figure 3 presents the effects of the SOP, $p_{so}$, and the COP, $p_{co}$, versus the eavesdropper density $\lambda_{E}$ with the different frequency. As $\lambda_{E}$ increases, we see that the $p_{so}$ keeps increasing and the $p_{co}$ remains constant for given a power *P*. In particular, compared with the 73 GHz band, the 28 GHz band reduces the $p_{co}$ and improves the reliability of the system, but at the same time increases the $p_{so}$ and reduces the secrecy performance of the system. These observations can help the system designer to select different frequency bands according to the actual performance requirements. For example, when the actual system requires high reliability, it is suitable to select the 28 GHz band.

**Figure 4:**
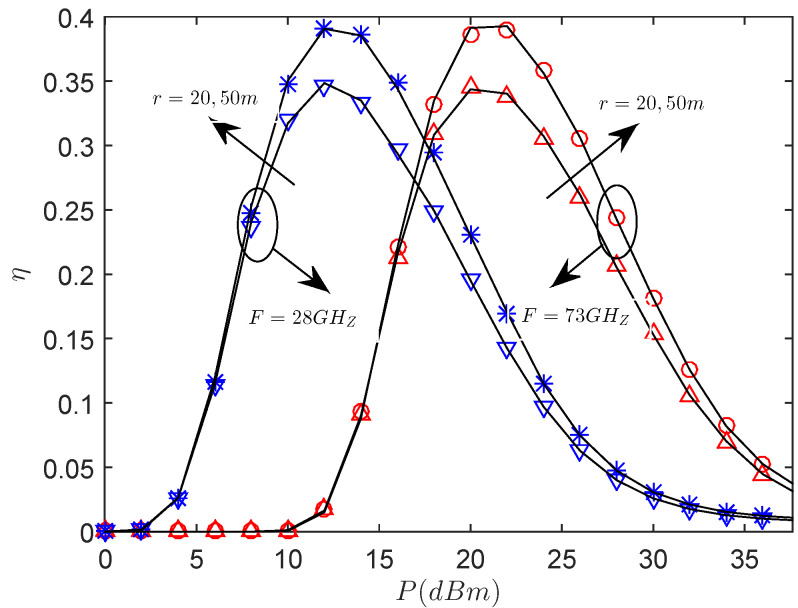
The η versus *P* with $R_{B}=1.5$ bps/Hz, $R_{S}=0.5$ bps/Hz, $\theta_{S}=\frac{\pi }{3}$, $\lambda_{E}=0.0002$ nodes/m${}^{2}$, $m_{S}=0.1$ and $M_{S}=200$.

Figure 4 presents the effects of the η versus the transmit power with the different sector secrecy guard zone radius *r* and frequency. We see that there exists an optimal *P* for maximizing the secrecy throughput at the considered mmWave frequencies. At a low transmission power region, the secrecy throughput of 28 GHz is better, and the same result can be achieved at 73 GHz when the transmission power becomes sufficiently large. The reason is that, in the case of low transmission power regime, mmWave link at lower mmWave frequencies experiences lower path loss and has stronger signal strength, thus achieving better performance. However, in the high transmission power regime, due to the high path loss at higher mmWave frequencies, the interference received by the legitimate user becomes lower, and the signal strength of the eavesdropper is also reduced at higher mmWave frequencies. In addition, we observe that the secrecy throughput of r=50 m is always superior to that of r=20 m. The reason is that there does not exist an eavesdropper within the sector secrecy guard zone when transmitting, and the secrecy performance becomes better with *r* increasing. Again, for achieving the same secrecy throughput, the transmit power required at 28 GHz is lower than 73 GHz.

**Figure 5:**
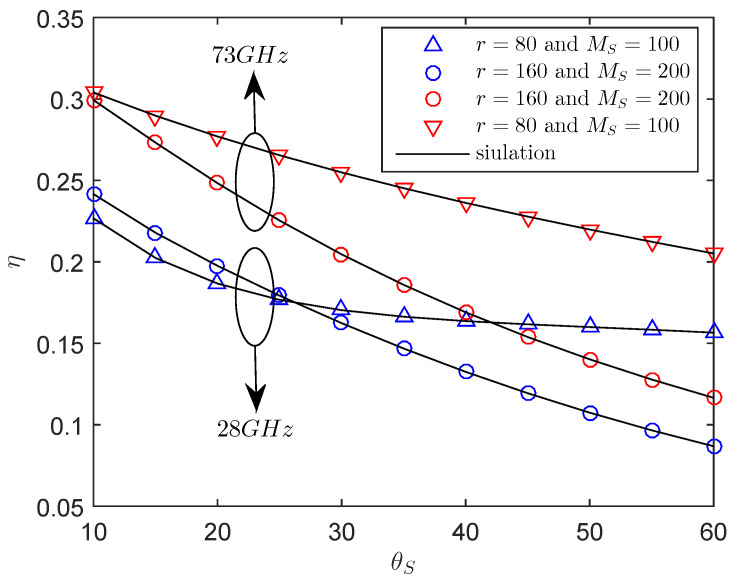
The η versus $\theta_{S}$ with $R_{B}=1.5$ bps/Hz, $R_{S}=0.5$ bps/Hz, P=30 dBm, μ=0.6, $\lambda_{E}=0.0002$ nodes/m${}^{2}$, $m_{S}=0.1$.

Figure 5 presents the central angle $\theta_{S}$ of sector secrecy guard zone on the secrecy throughput. It is obvious that the secrecy throughput of the system decreases with the central angle $\theta_{S}$ of the sector secrecy guard zone when the transmit power and the power allocation factor are sufficiently large. Specifically, under the same conditions, the performance of 73 GHz is superior to that of 28 GHz, which is mainly due to the difference path loss [45]. In addition, in the larger central angle region, the smaller radius is better than the larger radius due to fact that the larger sector secrecy guard zone may contain more eavesdroppers, which is detrimental to the secrecy performance.

**Figure 6:**
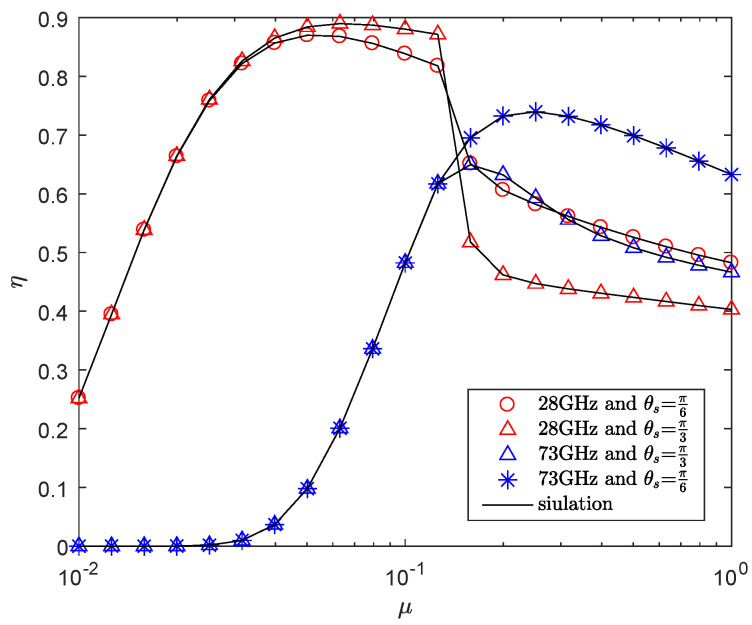
The η versus μ with $R_{B}=1.5$ bps/Hz, $R_{S}=0.5$ bps/Hz, P=30 dBm, $\lambda_{E}=0.0002$ nodes/m${}^{2}$, r=50 m, $m_{S}=0.1$ and $M_{S}=200$.

Figure 6 presents the effects of transmit power allocation factor on the secrecy throughput with the different frequency. We note that there exists an optimal transmit power allocation factor μ to maximize the secrecy throughput. When the μ is very small, it means that almost all power is allocated to AN, and the secrecy throughput is very small. With the increase of the power allocated to the information signal, that is, the increase of the power allocation factor, the secrecy throughput increases gradually. However, when the power allocation factor increases to a certain value, the secrecy throughput starts to accelerate drop, the reason is that when the power allocation factor is increased to a certain value, the power used to AN decreases, which increases the possibility that an eavesdropper can intercept information, and the security cannot be guaranteed. Therefore, more power should be allocated to the AN to interfere with the eavesdropper, which demonstrates that it is very important to set the power allocation of the AN and the information signal properly. In addition, when the power allocated to AN is reduced to a certain value, the attenuation of η in the wider main lobe is faster, which is because more eavesdroppers may be located in the wider sector secrecy guard zone. Again, for the same circumstance, when the power allocation factor is increased to a certain value, the secrecy throughput reaches the maximum value, and the performance of 73 GHz is better than that of 28 GHz with the further increase of power allocation factor for a given *r*.

**Figure 7:**
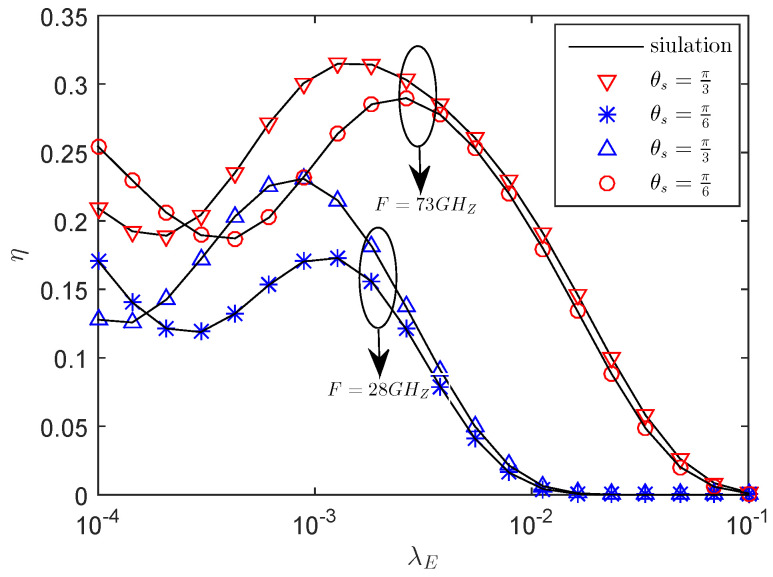
The η versus $\lambda_{E}$ with $R_{B}=1.5$ bps/Hz, $R_{S}=0.5$ bps/Hz, *P* = 35 dBm, *r* = 50 m, μ = 0.4, $m_{S}=0.1$ and $M_{S}=200$.

Figure 7 presents the effects of the η versus the eavesdropper density $\lambda_{E}$ with the different central angle $\theta_{S}$. We see that, when increasing of $\lambda_{E}$, the secrecy throughput declines. This can be explained by when $\lambda_{E}$ is low, the eavesdroppers located in $E\in \Phi_{1}$ and $E\in \Phi_{2}$ are indeed harmful for secrecy. However, as $\lambda_{E}$ grows large, the secrecy throughput increases. This is because, in this case, the eavesdropper will be in the sector secrecy guard zone, the transmitter emits AN to interfere with the eavesdropper, and the secrecy performance will be improved, which shows that AN can improve the secrecy throughput of the system. If $\lambda_{E}$ further increases, the secrecy throughput starts to decrease; the reason is that, as the number of eavesdroppers in the sector secrecy guard zone increases, the wiretapping capability of eavesdroppers increases, which deteriorates the secrecy performance. Obviously, when eavesdroppers exist in the sector secure region, transmitting AN is effective, but there is an appropriate $\lambda_{E}$, which makes the secrecy throughput reach the maximum. This shows that increasing the density of eavesdroppers not always deteriorates the secrecy performance. In this case, the large sector secrecy guard zone is superior to the small one.

**Figure 8:**
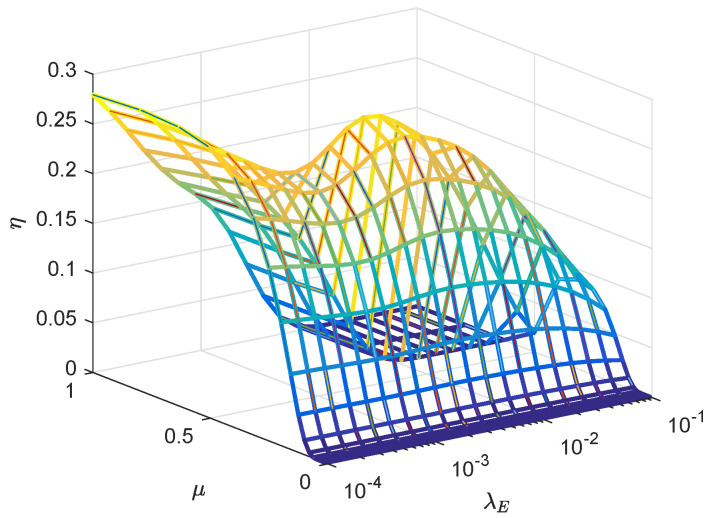
The η versus μ, $\lambda_{E}$ with $R_{B}=1.5$ bps/Hz, $R_{S}=0.5$ bps/Hz, *P* = 30 dBm, *F* = 28 GHz, *r* = 50 m, $\theta_{S}=\frac{\pi }{6}$, $m_{S}=0.1$ and $M_{S}=200$.

Figure 8 presents the effects of transmit power allocation factor μ and the eavesdropper density $\lambda_{E}$ on the secrecy throughput. From the simulation results, it shows that there exists an optimal transmit power allocation factor μ for maximizing the secrecy throughput with the changing $\lambda_{E}$. On the other hand, when the power allocation factor is smaller, no matter how high $\lambda_{E}$ is, the connection is interrupted, and the reliability of the system is not guaranteed. As both μ and $\lambda_{E}$ are sufficiently high, the secrecy outage occurs and the security is not guaranteed. It reveals that the power allocation of the information signal and AN need to be properly set depending on different system parameters for increasing the secrecy throughput.

**Figure 9:**
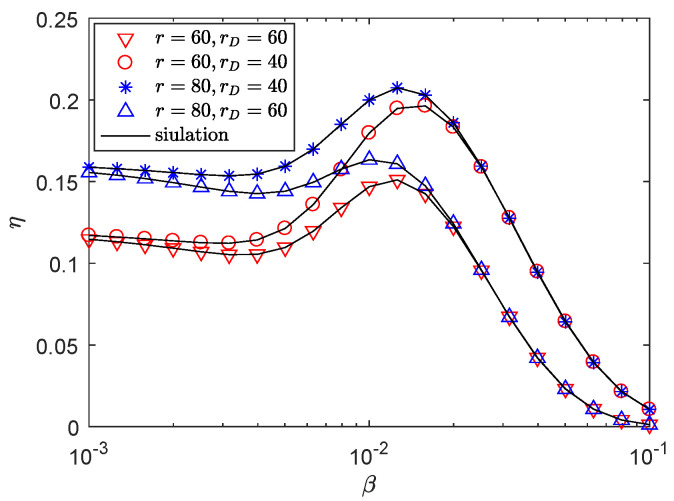
The η versus β, with $R_{B}=1.5$ bps/Hz, $R_{S}=0.5$ bps/Hz, *P* = 35 dBm, *F* = 28 GHz, $\lambda_{E}$ = 0.0002 nodes/m${}^{2}$, $\theta_{S}=\frac{\pi }{6}$, μ = 0.6, $m_{S}=0.1$ and $M_{S}=200$.

Figure 9 presents the effects of the η versus the blockage density β with different $r_{D}$. We observe that the secrecy throughput of r=80 m is always superior to that of r=60 m under the same system parameter settings. The reason is that, with the increase of *r*, if eavesdroppers exist in the sector secrecy guard zone and there is an appropriate $\lambda_{E}$, the transmitter will transmit AN interference to eavesdroppers, so the secrecy performance will be enhanced. In addition, increasing blocking intensity β does not always result in a strict decline in the secrecy throughput of mmwave wiretap networks. This shows that blockage plays an important role in the transmission of mmWave, which can be utilized to improve secrecy performance. From Figure 9, there exists an optimal β for maximizing the secrecy throughput at the different *r*. It is a meaningful conclusion that choosing sector secrecy guard zone according to the density of physical barriers and the distance of receiver can improve the secrecy throughput. As we improve β to the optimal point, the secrecy throughput attenuates, as NLOS communication dominates mmwave wiretap networks, using the multipath signals at the receiver. However, when the environment is full of physical obstacles, it is highly difficult for the message to reach the receiver, thus the secrecy throughput is gradually declining.

## 6. Conclusions

In this paper, we investigated secrecy performance under the Nakagami fading channel in an mmWave wiretap network. Then, an adaptive transmission scheme according to the locations of exavesdroppers is adopted for secrecy transmission, and we derived the SOP, COP and secrecy throughput under stochastic geometry. Specifically, there exists an optimal transmission power for the direct transmission and an optimal power allocation factor for the AN-assisted transmission by maximizing secrecy throughput. When the system parameters are set properly, AN can improve the secrecy throughput of the system. We got a meaningful conclusion that choosing sector secrecy guard zone with a larger radius according to the density of physical barriers and the distance of receiver can improve the secrecy throughput. In addition, it provides an important perception into the interaction among the transmitting power, main-lobe gain and the mmWave frequency. In future works, complex scenarios such as imperfect CSI, base-station (BS) cooperation and nonorthogonal multiple access (NOMA) will be considered. Furthermore, the results presented here can be combined with unmanned aerial vehicle (UAV) to analyze secrecy transmission capability.
